# Treatment of Diarrhea

**Published:** 1901-01

**Authors:** 


Treatment of Diarrhea.—In general the best treatment for the usual forms of transient diarrhea is, of course, the use of the well-known intestinal antiseptics as hydrochloric acid, creosote, salol or, best of all, benzosol. It should not be forgotten by the physician, however, that many times a serous diarrhea is not readily amenable to such drugs and that the rational treatment, after there have been enough passages to free the intestinal canal fairly from any offending material that may have been the exciting cause of the attack, consists in the use of small doses of opium, preferable in the form of Dover's powder—say 2½ grains—repeated until the excessive watery purging is checked. The opium stops the exhausting serous discharge and also supports the system against the exhaustion which is so marked a feature of this form of diarrhea. The patient need not know he is taking opium and its use is indicated for only two or three days.—*Cleveland Jour. of Med.*

